# Myc-like transcriptional factors in wheat: structural and functional organization of the subfamily I members

**DOI:** 10.1186/s12870-019-1639-8

**Published:** 2019-02-15

**Authors:** Ksenia V. Strygina, Elena K. Khlestkina

**Affiliations:** 1 Siberian Branch of the Russian Academy of Sciences, Institute of Cytology and Genetics, Lavrentjeva Ave. 10, Novosibirsk, 630090 Russia; 2 N.I. Vavilov All-Russian Research Institute of Plant Genetic Resources (VIR), Bolshaya Morskaya Str., 42-44, St. Petersburg, 190000 Russia

**Keywords:** Anthocyanin biosynthesis, bHLH, Flavonoid biosynthesis, Gene duplication, Myc, Osmotic stress, Salinity stress, Stress response, Transcription factor, *Triticum*, Wheat

## Abstract

**Background:**

Myc-like regulatory factors carrying the basic helix–loop–helix (bHLH) domain belong to a large superfamily of transcriptional factors (TFs) present in all eukaryotic kingdoms. In plants, the representatives of this superfamily regulate diverse biological processes including growth and development as well as response to various stresses. As members of the regulatory MBW complexes, they participate in biosynthesis of flavonoids. In wheat, only one member (*TaMyc1*) of the Myc-like TFs family has been studied, while structural and functional organization of further members remained uncharacterized. From two Myc-subfamilies described recently in the genomes of Triticeae tribe species, we investigated thoroughly the members of the subfamily I which includes the *TaMyc1* gene.

**Results:**

Comparison of the promoter regions of the *Myc* subfamily I members in wheat suggested their division into two groups (likely homoeologous sets): *TaMyc-1* (*TaMyc-A1*/*TaMyc1*, *TaMyc-B1*, *TaMyc-D1*) and *TaMyc-2* (*TaMyc-A2* and *TaMyc-D2*). It was demonstrated that the *TaMyc-D1* copy has lost its functionality due to the frame shift mutation. The study of functional features of the other four copies suggested some of them to be involved in the biosynthesis of anthocyanins. In particular, *TaMyc-B1* is assumed to be a co-regulator of the gene *TaC1-A1* (encoding R2R3-Myb factor) in the MBW regulatory complex activating anthocyanin synthesis in wheat coleoptile. The mRNA levels of the *TaMyc-A1*, *TaMyc-B1*, *TaMyc-A2* and *TaMyc-D2* genes increased significantly in wheat seedlings exposed to osmotic stress. Salinity stress induced expression of *TaMyc-B1* and *TaMyc-A2*, while *TaMyc-A1* was repressed.

**Conclusions:**

The features of the structural and functional organization of the members of subfamily I of Myc-like TFs in wheat were determined. Myc-like co-regulator (*TaMyc-B1*) of anthocyanin synthesis in wheat coleoptile was described for the first time. The Myc-encoding genes presumably involved in response to drought and salinity were determined in wheat. The results obtained are important for further manipulations with *Myc* genes, aimed on increasing wheat adaptability.

**Electronic supplementary material:**

The online version of this article (10.1186/s12870-019-1639-8) contains supplementary material, which is available to authorized users.

## Background

Most biological processes in eukaryotic cells are under the control of transcription factors (TFs). Each family of transcription factors is characterized by unique highly conserved domains [1, 2]. TFs usually contain two functional domains: one is associated with DNA binding, and the other – with protein dimerization [3]. In 1989, Murre, McCaw and Baltimore discovered that *MyoD* gene sharing a conservative cDNA region with members of *myc* family and the Drosophila *daughterless* (*da*), *achaete-scute* and *twist* gene family [4]. It was found that this conservative region encodes a protein motif, which is needed for DNA-binding and dimerization. These TFs were combined into one superfamily named bHLH (basic helix-loop-helix). It is the second largest family of TFs, which appeared in eukaryotic cells before the divergence of plants and animals [5]. The bHLH proteins are characterized by a highly conserved domain, which is approximately 60 amino acids in length [6, 7]. The bHLH domain is divided into two regions: the basic region and the helix-loop-helix (HLH) region. The basic region is approximately 15 amino acids in length and typically includes six basic residues, which function is binding with the palindromic hexanucleotide DNA region E-box (CANNTG), such as the G-box (CACGTG), in promoters of the target genes. The HLH region contains two amphipathic α-helices bound by a variable-length loop [6, 7]. The HLH promotes protein-protein interaction and acts as a region of homo- and heterodimerization. It was reported that bHLHs are key regulatory components in transcription networks that control a number of biological processes [5–7]. In plants, bHLH proteins are involved in the response to injury, drought and salinity stress, regulation of seed germination, trichome and fetal development, biosynthesis of uncolored flavonoids and their colored derivatives anthocyanins in flowers, leaves and fruits [6–16]. Transcriptional regulation of the flavonoid biosynthesis pathway has been extensively studied in many plant species, including maize (*Zea mays* L.), Arabidopsis (*Arabidopsis thaliana* L.) and grape (*Vitis vinifera* L.) [10, 17, 18]. In the biosynthetic pathway, bHLH proteins interact with members of other TFs families such as R2R3-MYB and WD40. Together they can form MBW complexes, which have been described in plants only [7, 10, 15, 16, 18]. The specificity of the MYB and bHLH proteins determines genes to be activated. The non-specific WD40 protein plays a more general role in the MBW regulatory complex.

Allohexaploid bread wheat (*Triticum aestivum* L., genome BBAADD, 2n = 6x = 42,) is one of the most important cereal crops. The bHLH-coding gene *TaMyc1*/*TaMyc1.1* (chromosome 2AL) controlling the synthesis of anthocyanins in the pericarp of wheat grains was previously isolated and characterized [19]. In addition, four more highly homologous copies of this gene were identified in homoeologous group 2 chromosomes of bread wheat. These genes form a cluster with orthologous sequence *HvAnt2*/*HvMyc1* (2HL) from barley (HH, 2n = 14, *Hordeum vulgare* L.), which also regulates anthocyanin pigmentation in pericarps [19–23]. Later, by searching for homologous sequences, six highly homologous *Myc*-like sequences were identified and annotated in wheat genome (five copies located on the long arms of in homoeologous group 4 chromosomes and one copy located on 2DL chromosome) [24]. Also, it was found that in barley genome there is one *Myc*-like gene copy located on 4HL controlling anthocyanin biosynthesis in barley aleurone layer [25]. However, functions of *Myc* gene copies in wheat genome with the exception of *TaMyc1* remain unknown. In the current study, we investigated features of structural and functional organization of further members of the Myc-like TFs’ family in wheat, exploring their relation to anthocyanin synthesis and stress response. We also tested, whether methylation status of *TaMyc1* promoter could play a role in diverse activity of different alleles of this gene.

## Results

### Coding sequences of the *Myc* genes

**Fig. 1:**
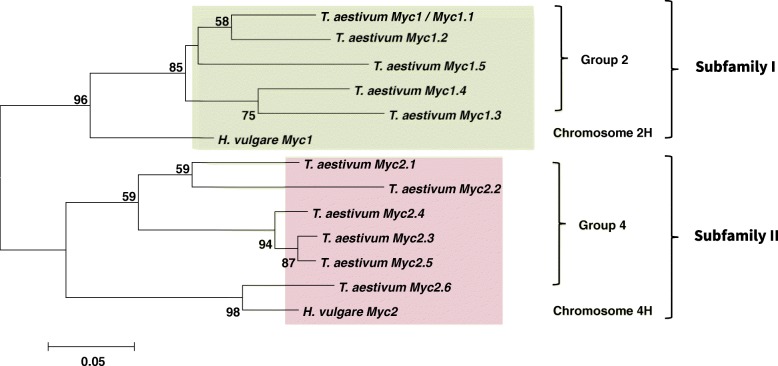
Genetic similarity of *Myc*-like genes (bHLH motif). Phylogenic tree was constructed in MEGA 7.0 with Neighbor-Joining method with 1000 bootstrap replicates. Green colour – homoeologous group 2 chromosomes genes (subfamily I). Pink colour – homoeologous group 4 chromosomes genes (subfamily II)

Genetic relationship between 11 bread wheat and 2 barley *Myc* genes was established using a Neighbor-Joining phylogenetic analysis of their full-length bHLH domains. The results of the analysis demonstrated existence of two Myc-subfamilies in Triticeae tribe (Fig. 1), originating from the ancient duplication, which involved chromosomes 2 (carrier of the *Myc* copy giving rise to subfamily I) and 4 (subfamily II). Subfamily II included recently discovered *HvMyc2* gene [25].

**Fig. 2:**
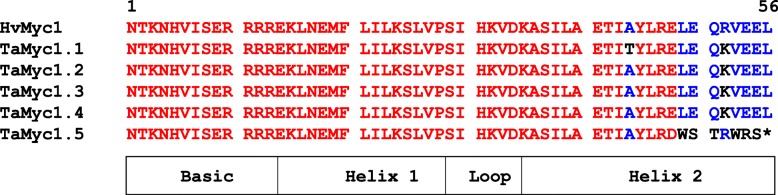
The multiple alignment of the Myc subfamily I proteins. Analysed protein motif is Myc-type, basic helix-loop-helix (bHLH) domain (IPR011598). Multiple sequence alignment was performed using MultAlin program. Red - high consensus, blue - low consensus, black - neutral

The *TaMyc1.5* gene carries the single nucleotide deletion in the functionally significant bHLH domain (Fig. 2). This mutation should lead to the formation of non-functional bHLH domain, since it leads to both functional amino acid replacement and to the premature appearance of a stop codon in the Helix 2 region of bHLH domain. The presence of this mutation in the genome of bread wheat was confirmed by resequencing of the *TaMyc1.5* gene in the cultivars Chinese Spring and Saratovskaya 29. Furthermore, the subsequent analysis of *Myc1.5* from *Aegilops tauschii* L. (DD, 2n = 14, donor of the D-genome of bread wheat) revealed the same mutation that indicates the occurrence of this deletion and formation of a non-functional gene at a diploid level (contig 1,770,231, URGI database). This nonfunctional *TaMyc1.5* copy was not included in further experiments aimed on comparison of transcriptional activity of the subfamily I members.

### Promoter sequences of the *Myc* genes (subfamily I)

**Fig. 3:**
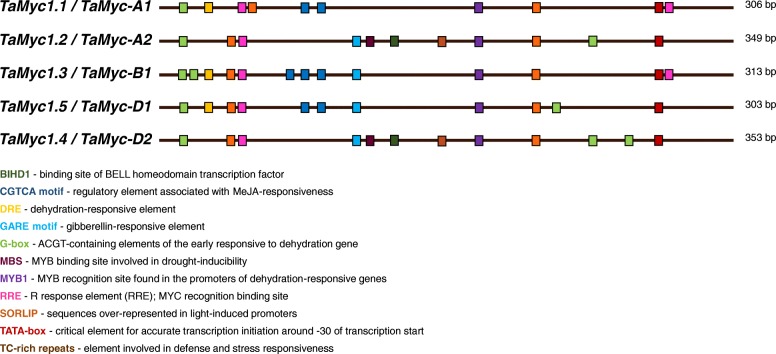
Diverse promoter structure of the Myc subfamily I genes in wheat. Promoter analysis was performed using New PLACE database. Each square represents a corresponding colour motif

Based on promoter sequence of *TaMyc1*/*TaMyc1.1* (GenBank: KJ747954) from the NCBI database, sequence alignment was applied to reveal promoter regions of *Myc* genes from homoeologous group 2 chromosomes (~ 350 bp from transcriptional start site of *TaMyc1*). The multiple sequence alignment as well as analysis using the New PLACE database (for detection of individual elements and their position relative to each other) showed that the genes are divided into two groups due to the features of their promoter regions and phylogenic relationship: *TaMyc1.1*, *TaMyc1.3*, *TaMyc1.5* (the 1st group) and *TaMyc1.2*, *TaMyc1.4* (the 2nd group) (Fig. 3, Additional file 1, Additional file 2). The sequences within each group have retained a common set of regulatory elements such as transcription factors binding sites, stress-responsive elements and sites of light-induced transcription activation (Fig. 3, Additional file 3). We suggested that common sequence patterns could be related with common origin of homoeologous genes as evidenced by phylogenetic analysis of the promoter sequences (Additional file 2). Based on rules of designation of homoeologous genes in wheat, the *Myc* subfamily I members were re-designated: *TaMyc1.1* (*TaMyc1*) *– TaMyc-A1*, *TaMyc1.2 – TaMyc-A2*, *TaMyc1.3 – TaMyc-B1*, *TaMyc1.4 – TaMyc-D2*, *TaMyc1.5 – TaMyc-D1*, respectively (Fig. 3).

### Expression of the *Myc* genes in the coleoptile of differently colored wheat cultivars

**Fig. 4:**
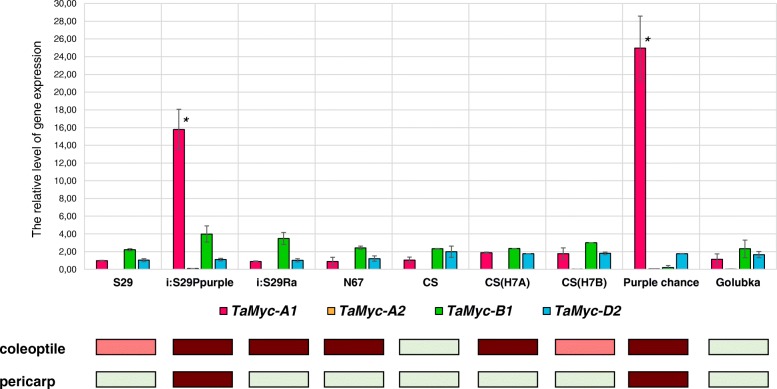
The expression of the *Myc* subfamily I genes in the coleoptile of wheat genotypes having different coloration (the 5th day after germination). The data are presented as mean value ± standard error. *differences are statistically significant between coloured genotypes and S29 at *p* ≤ 0.005 (*U*-test)

Previously, the *TaMyc-A1* gene was shown to be involved in anthocyanin biosynthesis in wheat pericarp, while other subfamily I members were inactive in this tissue [19]. To explore whether they could be related to anthocyanin synthesis in other parts of wheat plant, we performed qRT-PCR with gene-specific primers to *TaMyc-A1*, *TaMyc-A2*, *TaMyc-B1* and *TaMyc-D2* in coleoptile of wheat cultivars and lines having green (uncolored), light red and dark red coleoptile coloration (Table 1, Additional file 4). The *TaMyc-A1* expression on 5th day after seed germination was much higher (almost in 8–15 and 13–25 times, respectively) in coleoptiles of two genotypes i:S29*Pp-A1Pp-D1Pp3*^P^ and Purple chance (carriers the dominant allele of *TaMyc-A1*) in comparison with all other genotypes (Fig. 4). A high level of *TaMyc-A1* expression could explain the appearance of a dark red colour of coleoptile in i:S29*Pp-A1Pp-D1Pp3*^P^ and Purple chance. However, this gene cannot be considered the main regulator of anthocyanins accumulation in coleoptile, since the lines with a dark red colour of coleoptile (i:S29*Ra*, CS(Hope 7A), Novosibirskaya 67) did not share the same *TaMyc-A1* expression level. Overall, qRT-PCR at the 5th day after germination revealed no correlation between relative level of gene expression and the presence of anthocyanins in wheat coleoptile (Fig. 4), therefore we carried out the analysis in dynamics from the 2nd till the 5th day after germination, using 3 genotypes: uncolored CS, light-red S29 and dark-red CS(H7A) (Table 1).

**Table 1:** Genetic stocks of wheat genotypes used in the current study, and their phenotypic characteristics. NIL – near-isogenic line, SCSL – single chromosome substitution line

Cultivar name	Description	Coleoptile coloration	Pericarp coloration
Saratovskaya 29 (S29)	Russian spring wheat	light red color	uncolored
i:S29*Pp-A1Pp-D1Pp3*^P^	Wheat NIL developed on S29, donor Purple [40, 41]	dark red color	dark purple color
i:S29*Ra*	Wheat NIL developed on S29, donor Ulyanovka [40, 42]	dark red color	uncolored
Novosibirskaya 67	Russian spring wheat	dark red color	uncolored
Chinese Spring (CS)	Chinese spring wheat	uncolored	uncolored
CS(Hope 7A)	Wheat SCSL developed on CS, donor Hope [43]	dark red color	uncolored
CS(Hope 7B)	Wheat SCSL developed on CS, donor Hope [43]	light red color	uncolored
Purple chance	Russian spring wheat [44]	dark red color	dark purple color
Golubka	Russian spring wheat	uncolored	uncolored

**Fig. 5:**
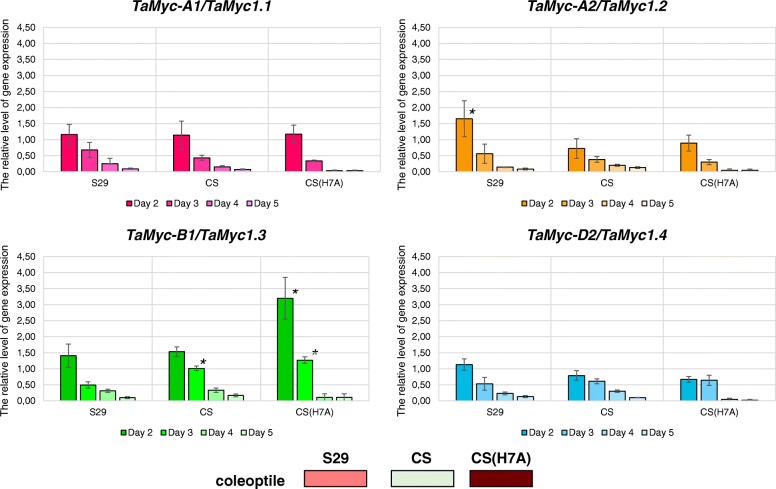
The expression of the *Myc *subfamily I genes in the coleoptile during wheat seedling development (from the 2nd to the 5th day). Selected genotype: sister lines CS (uncolored) and CS(H7A) (dark-colored) and the unrelated genotype S29 (light-coloured). The data are presented as mean value ± standard error. *differences are statistically significant between coloured genotypes and S29 at *p* ≤ 0.05 (*U*-test)

For all of the analyzed genes in each of the three genotypes we found a general trend of decrease of the mRNA levels from very high at the 2nd day to extremely low at the 5th day (Fig. 5). We found statistically significant changes in the expression levels of *TaMyc-B1* gene between the sister lines CS and CS(H7A) at the early stages of development (Fig. 5). These genotypes differ by the allelic state of the *TaC1-A1* gene, which is considered to be the Myb-encoding regulator of the anthocyanin synthesis in coleoptile. The introduction of the dominant *TaC1-A1* gene into the CS genome causes an increase of the *TaMyc-B1* gene expression 2-fold (Fig. 5). We assumed that *TaMyc-B1* could be the main co-regulator of *TaC1-A1* in anthocyanin biosynthesis control in wheat coleoptile. In addition, for the S29 genotype, it was found that the expression of the *TaMyc-A2* gene on the 2nd day of development was significantly higher than its expression in CS and CS(H7A) (approximately 2-fold higher). This difference may be the reason for the appearance of a weak anthocyanin colour of S29 coleoptile (Table 1, Additional file 4). S29 is a carrier of another *TaC1-A1* allele differing from both CS and CS(H7A). Specific recognition of different *TaMyc-1* copies by different R2R3-*Myb* factors can be hypothesized for future verification.

### Methylation patterns of the *TaMyc-A1 *promoter

**Fig. 6:**
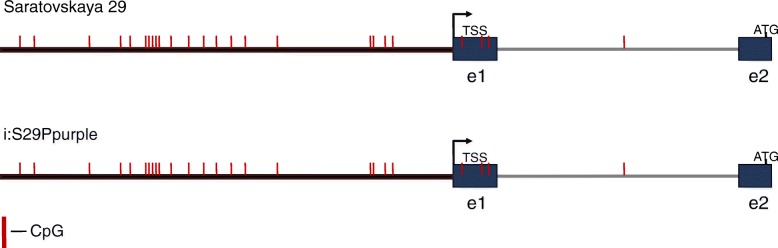
Methylation patterns of the *TaMyc-A1* promoter regions in the pericarp of isogenic lines differing in anthocyanin pigmentation of this tissue. ATG – the triplet code for the first amino acid methionine, e – exon, TSS – transcription start site

Due to the detection of increased transcriptional activity of the *TaMyc-A1* gene in genotypes with anthocyanin pericarp pigmentation (Fig. 4), we analyzed the DNA methylation in promoter of this gene by bisulfite sequencing in genotypes of near isogenic lines (NILs), differing in allelic state of *TaMyc-A1*: lines S29 and i:S29*Pp-A1Pp-D1Pp3*^P^ (Table 1). As a result of the analysis, we showed that the analyzed 748 bp region contains methylated sites neither in the promoter region (406 bp) nor in 342 bp from the transcription start site in CpG and plant-specific non-CpG methylation sites (Fig. 6, Additional file 5). Thus, we assume that the level of expression of the regulatory *TaMyc-A1* gene is determined by the structure of the *cis*-regulatory components of the gene. Such epigenetic mechanisms as DNA methylation apparently do not affect the activity of the analyzed gene.

### Stress response relation of *Myc *genes

**Fig. 7:**
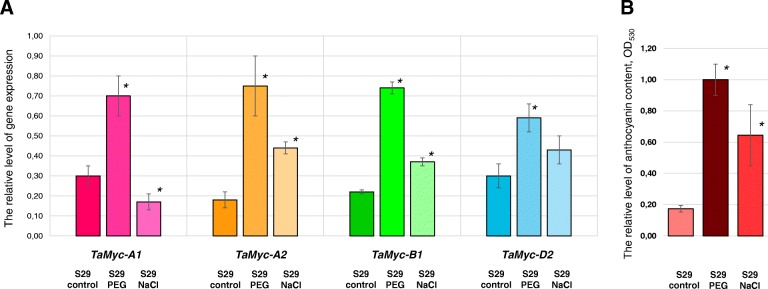
**a** The expression of the *Myc*-like genes in wheat coleoptiles under salinity and drought stress (the 4th day after germination). **b** The relative level of anthocyanin content under the same conditions. The data are presented as mean value ± standard error. *differences are statistically significant between stressed and control S29 at *p* ≤ 0.05 (*U*-test)

Finding the stress-dependent elements in the promoters of the *Myc* subfamily I genes (Fig. 3, Additional file 3) was the reason for further analysis of their expression levels in response to drought and salinity stress. The relative levels of the *Myc* genes mRNA were measured in coleoptiles of S29 plants in response to either 10% PEG or 0.2 M NaCl and was compared to that in plants germinated in distilled water. The mRNA levels of the *TaMyc-A1*, *TaMyc-B1*, *TaMyc-A2* and *TaMyc-D2* genes increased significantly in wheat seedlings exposed to 10% PEG. Salinity stress induced expression of *TaMyc-B1* and *TaMyc-A2* only. The *TaMyc-A1* mRNA level was decreased, while *TaMyc-D2* did not respond to 0.2 M NaCl treatment. The changes of the mRNA levels in response to stress correlated with the changes of anthocyanin content in the coleoptile with the exception of *TaMyc-A1* (Fig. 7a, and b).

## Discussion

Polyploids have many advantages over diploid ancestors. The evolution of genes and genomes of polyploid organisms remains a subject of extensive research in the field of evolutionary biology. Organisms acquire new functions due to gene duplications such as resistance to diseases and adaptation to stress and extreme environmental conditions [26, 27]. Most duplicated genes remain active in neopolyploid organisms, contributing to the beneficial effect of an additional gene dose. For example, polyploidization of wheat, cotton and soybean has contributed to the improvement of such important agronomical traits as grain quality and flowering time [26]. Bread wheat *T. aestivum* is a hexaploid species which was formed as a result of hybridization between tetraploid *Triticum turgidum* (2n = 4x = 28, BBAA) and diploid *Aegilops tauschii* (2n = 14, DD) about 8000 years ago [28–30]. Bread wheat genome carries both paralogous and orthologous (homoeologous) copies of many structural and regulatory genes, including Myc-encoding genes. Myc TFs are the members of the regulatory MBW complexes [7, 10, 18]. From 17 possible *Myc* copies potentially involved in regulation of flavonoid biosynthesis, *T. aestivum* genome retained 11 (6 copies were likely pseudogenized) [24]. The retention of the 11 copies may suggest their specialization in synthesis of different classes of flavonoids in different tissues. The specific features were determined previously for the *TaMyc-A1* gene, which together with the Myb-encoding *TaC1-D1* gene controls the biosynthesis of anthocyanins in the pericarp of wheat grains [16, 19]. The specific features of other 10 copies remained unknown. We have found that at least two more *Myc* copies (*TaMyc-A2* and *TaMyc-B1*) could be involved in the biosynthesis of anthocyanins (Fig. 5). In addition, we demonstrated that the early stage of wheat development (until the 3rd day after germination) may be a key stage for initiation of the anthocyanin biosynthesis (Fig. 5). Probably, therefore we did not detect any significant differences in the *Myc* genes expression pattern among the genotypes differing by the coleoptile coloration at the later stage (5th day after the germination; Fig. 4).

It is known that after duplication two identical gene copies are most often redundant. Duplicated copies of the gene usually undergo one of the possible evolutionary events: pseudogenization (one of the duplicated genes becomes nonfunctional), neofunctionalization (one of the duplicated genes may acquire a new function) or subfunctionalization (the copies can share the functions of the original gene) [31]. While the above mentioned *TaMyc-A1* or *TaMyc-B1* gene may represent the examples of subfunctionalization ([19]; Fig. 5), the *TaMyc-D1* gene is undergoing pseudogenization due to a single nucleotide frame shift mutation detected (Fig. 2). Increasing the dose of some genes may have adverse consequences. In such cases, the normalization of the gene dose occurs owing to genetic and epigenetic changes. For example, among three homoeologous copies of the *WLHS-1* wheat gene one gene has lost it functionality due to mutation in the functional domain, while another copy became silent because of hypermethylation, and only the third gene retained its functionality [32]. Such epigenetic factor as DNA methylation (mC) is significant for binding of TFs to the gene *cis*-regulatory region (TF recognition elements usually present in the promoter region and the first intron) [33–35]. The epigenetic changes are potentially reversible and less stable than genetic changes. DNA methylation is a covalent modification of cytosine, which predominantly occurs on the CpG dinucleotide in plants and animals. However, for plants, DNA methylation is observed not only at CpG sites, but also in CpHpG and CpHpH sites, where H is adenine, cytosine or thymine [33]. In wheat, epigenetic changes can be potentially found among both alleles of the same gene or homoeoalleles. Since promoter regions of the *TaMyc-A1* (*TaMyc1*) dominant (transcribed in pericarp) and recessive (non-transcribed in pericarp) alleles were identical [19], the hypothesis was proposed about putative difference in the methylation patterns of the *cis*-regulatory regions of the *TaMyc-A1* alleles. However, the hypothesis was not confirmed (Fig. 6). Finally, the difference between the alleles has been found in copy number variation of the 261 bp-element upstream the promoter [16, 36]. Tandem duplication of this element results in strong activation of *TaMyc-A1* expression and appearance of anthocyanin pigment in wheat pericarp.

Pigmentation is only one of the multiple roles of flavonoid substances. They are also widely known for their adaptation properties, which help plant to survive in unfavorable environment conditions [37]. Comparative assessment of mRNA levels of the members of *Myc* subfamily I in optimal growth vs stress (salinity and drought) conditions suggested them presumably to be involved in a stress-dependent response (Fig. 7). Furthermore, the parallel was found between the quantitative content of anthocyanins in wheat coleoptile and the relative level of the *Myc* genes expression. The exception was the *TaMyc-A1* gene, for which a significant decrease of mRNA level in response to treatment with a 0.2 M NaCl solution was detected (Fig. 7). In plants, a wide range of evidences for the relationship between abiotic stress and flavonoid biosynthesis has been received. Drought and salinity cause such negative effects in the cell as osmotic and oxidative stress, repression of photosynthesis, damage to cellular components and metabolic dysfunction. Different flavonoid substances participate in osmoregulation, protection of photosynthetic apparatus and plasma membrane, scavenging free radicals, which appear during oxidative stress development [37]. We propose, that the *Myc* subfamily I genes may participate in improvement wheat plant tolerance under drought and salinity stress, due to activation of synthesis of various flavonoid compounds, including anthocyanins.

## Conclusions

The features of the structural and functional organization of the members of subfamily I of Myc-like TFs in wheat were determined. *Myc*-like co-regulator (*TaMyc-B1*) of anthocyanins synthesis in wheat coleoptile was described for the first time. The Myc-encoding genes involved in salinity and drought stress response were determined in wheat. The results obtained are important for understanding of (i) retaining multiple *Myc* copies in wheat genome, (ii) regulation features of flavonoid biosynthesis in wheat, as well as for (iii) further manipulations with *Myc* genes aimed on increasing adaptability of wheat plants.

## Methods

### Multiple sequence alignments, identification of conserved motifs and phylogenetic analysis

Wheat *Myc*-coding genes (promoter and coding sequences) were selected from the International URGI database (https://urgi.versailles.inra.fr) according [24] using BLAST. Barley genes *HvAnt2*/*HvMyc1* (GenBank: KX035100) and *HvMyc2* (GenBank: MF679157) were taken from the NCBI database (https://www.ncbi.nlm.nih.gov). Multiple sequence alignment was performed using MultAlin program (http://multalin.toulouse.inra.fr/multalin).Promoter sequences of *Myc* genes from subfamily I were predicted by comparison with *TaMyc-A1* (GenBank: KJ747954). Protein sequences were analyzed with InterPro program by predicting domains and important binding sites (https://www.ebi.ac.uk/interpro). Promoter analysis was performed using New PLACE database, containing *cis*-acting regulatory DNA elements of vascular plants (https://sogo.dna.affrc.go.jp/cgi-bin/sogo.cgi?lang=en&pj=640&action=page&page=newplace) (Additional file 3). A phylogenetic tree was constructed with MEGA 7.0 software (http://www.megasoftware.net) using the Neighbor-Joining method with 500 (promoter sequences) and 1000 (bHLH motif sequences) bootstrap replicates. The resulting images show bootstrap accounts ≥50%.

### Plant materials and growth conditions

Plant material included nine cultivars of the hexaploid wheat *T. aestivum* from the ICG collection GenAgro, contrasting by anthocyanin pigmentation in its coleoptile and pericarp. These cultivars and their phenotypes are presented in Table 1. Seeds for RNA extraction were germinated in the climatic chamber “Rubarth Apparate” (RUMED GmbH, Laatzen, Germany) on moist filter paper under a 12 h photoperiod at 20 °C. The germinated one-day old seedlings were exposed to 0 (control), 200 mM NaCl and 15% polyethylene glycol (PEG 6000) simulating salinity and drought stress, respectively. The experiments were conducted in triplicate for each stage of development, concentration and each genotype. The plants for DNA extraction from pericarps were grown using resources of ICG Greenhouse Core Facilities (Novosibirsk, Russia) under 12 h of light per day at 20–25 °C.

### Anthocyanins extraction and relative content measurements

Anthocyanins were extracted from the frozen and homogenized coleoptiles at the 4th day after germination in 1%-HCl/methanol according [38]. Anthocyanin extractions and measurements were performed in three replicates. The relative anthocyanin content was evaluated by spectrophotometry at 530 nm wavelength.

### DNA extraction and bisulfite treatment

Total genomic DNA was extracted from fresh wheat pericarps using the DNeasy Plant Mini Kit (QIAGEN) from two cultivars: S29 (uncolored pericarp) and i:S29*Pp-A1Pp-D1Pp3*^P^ (dark purple pericarp) (Table 1). Pericarps were scalpeled from grains at early dough stage maturity. One μg of gDNA from each sample was treated with sodium bisulfite using the EpiTect kit (QIAGEN).

### RNA isolation and cDNA synthesis

RNAs from coleoptile samples were extracted using the RNeasy Mini Kit (QIAGEN). All isolated RNAs were treated with the RNase-free DNase set (QIAGEN). Total RNA was converted to single-stranded cDNA in a 20-μL reaction from a template consisting of 0.5 μg of total RNA using the RevertAid First Strand cDNA Synthesis Kit (Thermo Fisher Scientific Inc.).

### Primer design and PCR amplification, sequencing

Gene-specific primers were developed: (1) for amplification partial gene sequences and full length CDS were designed using OLISO software; (2) for promoter region of *TaMyc-A1* gene using MethPrimer (Table 2). Amplification was made in 20 μL PCRs. Reaction mixtures contained 50–100 ng of genomic template DNA, 1 ng of each of primer, 0.25 mM of each dNTP, 1x reaction buffer (67 mM TrisHCl, pH 8.8; 2 mM MgCl_2_; 18 mM (NH_4_)_2_SO_4_; 0.01% Tween 20) and 1–2.5 U Taq polymerase. DNA templates were amplified with initial denaturation at 94 °C for 2 min, 35 cycles were run at 94 °C for 1 min, 50–63 °C for 1 min (Table 2), and 72 °C for 0.5–2 min, followed by a final extension at 72 °C for 5 min. PCR products were separated on agarose gels, stained with ethidium bromide and visualized under UV light. The amplified fragments were purified from an agarose gel using a DNA Clean kit (Cytokine). DNA sequencing was performed using the SB RAS Genomics core facilities (Novosibirsk, Russia). Obtained sequences were deposited in GenBank (NCBI).

**Table 2:** Gene-specific primers used for amplification of the *TaMyc* genes in current study. Primer pairs used for promoter region amplification of *TaMyc-A1* are underlined

Gene	Purpose	Annealing temperature. (°C)	PCR product length (bp) DNA/cDNA	Forward primer (5′ → 3′)	Reverse primer (5′ → 3′)
*TaMyc-A1*	qRT-PCR, sequencing	60	283/177	AACCATGTCATTTCGGAGAGGA	GGCCGCCCTGTTGGATC
	PCR, sequencing	60	−/1707	ATGGCGCTGCCAGTAGTTCGTC	TCATGGCCTGCGAATAGCTCTCT
	cloning	50	292/−	TTATTAGATTAAGTATAGTTTTTTTGGTAGAA	CAACATCAAAATACTATACTACACTAACA
	cloning	53	231/−	TGTTAGTGTAGTATAGTATTTTGATGTTG	ACCATAACCCATAAATAAAAAAAAAACTTCC
	cloning	57	309/−	GGAYGATAGGTTGGTTTTTGAGTTTTTTG	CCATTACCACACTATTTCCTTCCTTCA
*TaMyc-A2*	qRT-PCR, sequencing	60	334/220	AACCATGTCATTTCGGAGAGGA	TCTTCCCGCCGACTTCATGA
	PCR, sequencing	60	−/1697	ATGGCGCTGCCAGTAATTCGTC	TCAGCGCCTGCGTATAGCTTTCT
*TaMyc-B1*	qRT-PCR, sequencing	60	347/198	AACCATGTCATTTCGGAGAGGA	GAGTTTTCGGACGGCTGTTG
	PCR, sequencing	60	−/1692	ATGGCGCTGTCAGTAGTTCGTC	TCAGCGCCTGCGTATGGCTCT
*TaMyc-D1*	PCR, sequencing	60	504/244	GCTCATCGTGCTTTGCGG	GCCGCCCTGTTGGATTCT
*TaMyc-D2*	qRT-PCR, sequencing	60	341/188	AACCATGTCATTTCGGAGAGGA	ACGGCTGTTCCGGCCA
	PCR, sequencing	63	−/1665	ATGGCGCTGCCGGTAGTTCATC	TCATCCGGGTTCCACGGCTCCA

### Bisulfite genomic sequencing analysis

Amplified bisulfite PCR products for each samples described above were subcloned using the PCR Cloning Kit (Qiagen). Plasmid DNA of 10 insert-positive clones for each PCR product were sequenced in both directions with M13 primers.

### Quantitative RT-PCR analysis

qRT-PCR was performed with the primers from Table 2. A fragment of the *Ubiquitin* gene sequence was used for reference [39]. The amplifications were performed in an ABI Prism 7000 Sequence Detection System (Applied Biosystems) applying a SYBR Green I kit (Syntol). Pre-determined amounts of cloned cDNA were used to generate standard curves. Each sample was run in three technical replications. The differences among genotypes were tested by Mann-Whitney *U*-test, taking *p* ≤ 0.05 and 0.005 as significant.

## Additional files

Additional file 1: The multiple alignment of the *Myc* subfamily I genes. Multiple sequence alignment was performed using MultAlin program. Sequences were selected from the International URGI database according [24]. Red is high consensus colour, blue is low consensus colour, black is neutral colour. (PPTX 48 kb)

Additional file 2: Genetic similarity of promoter sequences of *Myc*-like genes subfamily I. Phylogenic tree was constructed in MEGA 7.0 with Neighbor-Joining method with 500 bootstrap replicates. Blue colour – the 1st group. Green colour – the 2nd group. (PPTX 37 kb)

Additional file 3: Putative cis-acting regulatory elements identified in the *Myc* promoters. Promoter analysis was performed using New PLACE database. “+” – coding strand, “–” – template strand*. (DOCX 27 kb)*

Additional file 4: Coleoptile colour of selected wheat samples at the fifth day after germination. (PPTX 459 kb)

Additional file 5: The results of bisulfite sequencing in genotypes of near isogenic lines, differ in allelic state of *TaMyc-A1*: lines S29 and i:S29*Pp-A1Pp-D1Pp3*^P^. First and second exons are marketed with yellow and green colour, respectively. Blue – unmethylated CpG sites, red – start-codon (ATG site). (DOCX 13 kb)

## Abbreviation

MBW: M – Myb, B – bHLH (Myc), W – WD40
