# Supplementary material for: Myc-like transcriptional factors in wheat: structural and functional organization of the subfamily I members
Source: BMC Plant Biol. 2019 Feb 15;19(Suppl 1):50. doi: 10.1186/s12870-019-1639-8 (PMC6393960; doi:10.1186/s12870-019-1639-8)
Supplement: Supplementary file 1 — The multiple alignment of the Myc subfamily I genes. Multiple sequence alignment was performed using MultAlin program. Sequences were selected from the International URGI database according [24]. Red is high consensus colour, blue is low consensus colour, black is neutral colour. (PPTX 48 kb) [file 12870_2019_1639_MOESM1_ESM.pptx]

## Slide 1
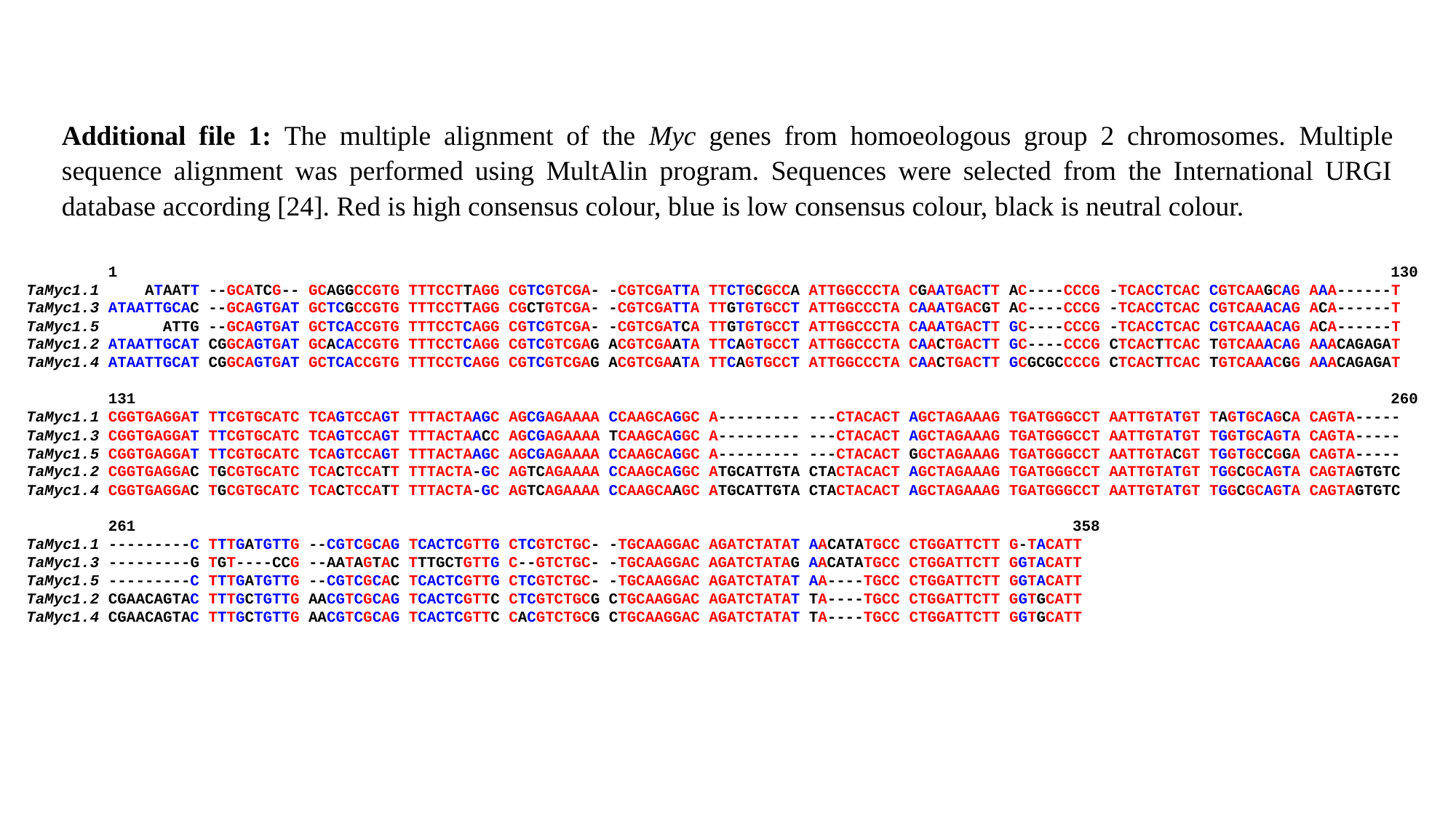

Additional file 1: The multiple alignment of the Myc genes from homoeologous group 2 chromosomes. Multiple sequence alignment was performed using MultAlin program. Sequences were selected from the International URGI database according [24]. Red is high consensus colour, blue is low consensus colour, black is neutral colour.
 1 												 130
TaMyc1.1 	 ATAATT --GCATCG-- GCAGGCCGTG TTTCCTTAGG CGTCGTCGA- -CGTCGATTA TTCTGCGCCA ATTGGCCCTA CGAATGACTT AC----CCCG -TCACCTCAC CGTCAAGCAG AAA------T
TaMyc1.3 ATAATTGCAC --GCAGTGAT GCTCGCCGTG TTTCCTTAGG CGCTGTCGA- -CGTCGATTA TTGTGTGCCT ATTGGCCCTA CAAATGACGT AC----CCCG -TCACCTCAC CGTCAAACAG ACA------T
TaMyc1.5 	 ATTG --GCAGTGAT GCTCACCGTG TTTCCTCAGG CGTCGTCGA- -CGTCGATCA TTGTGTGCCT ATTGGCCCTA CAAATGACTT GC----CCCG -TCACCTCAC CGTCAAACAG ACA------T
TaMyc1.2 ATAATTGCAT CGGCAGTGAT GCACACCGTG TTTCCTCAGG CGTCGTCGAG ACGTCGAATA TTCAGTGCCT ATTGGCCCTA CAACTGACTT GC----CCCG CTCACTTCAC TGTCAAACAG AAACAGAGAT
TaMyc1.4 ATAATTGCAT CGGCAGTGAT GCTCACCGTG TTTCCTCAGG CGTCGTCGAG ACGTCGAATA TTCAGTGCCT ATTGGCCCTA CAACTGACTT GCGCGCCCCG CTCACTTCAC TGTCAAACGG AAACAGAGAT
 131 											 260
TaMyc1.1 CGGTGAGGAT TTCGTGCATC TCAGTCCAGT TTTACTAAGC AGCGAGAAAA CCAAGCAGGC A--------- ---CTACACT AGCTAGAAAG TGATGGGCCT AATTGTATGT TAGTGCAGCA CAGTA-----
TaMyc1.3 CGGTGAGGAT TTCGTGCATC TCAGTCCAGT TTTACTAACC AGCGAGAAAA TCAAGCAGGC A--------- ---CTACACT AGCTAGAAAG TGATGGGCCT AATTGTATGT TGGTGCAGTA CAGTA-----
TaMyc1.5 CGGTGAGGAT TTCGTGCATC TCAGTCCAGT TTTACTAAGC AGCGAGAAAA CCAAGCAGGC A--------- ---CTACACT GGCTAGAAAG TGATGGGCCT AATTGTACGT TGGTGCCGGA CAGTA-----
TaMyc1.2 CGGTGAGGAC TGCGTGCATC TCACTCCATT TTTACTA-GC AGTCAGAAAA CCAAGCAGGC ATGCATTGTA CTACTACACT AGCTAGAAAG TGATGGGCCT AATTGTATGT TGGCGCAGTA CAGTAGTGTC
TaMyc1.4 CGGTGAGGAC TGCGTGCATC TCACTCCATT TTTACTA-GC AGTCAGAAAA CCAAGCAAGC ATGCATTGTA CTACTACACT AGCTAGAAAG TGATGGGCCT AATTGTATGT TGGCGCAGTA CAGTAGTGTC
 261 								 358
TaMyc1.1 ---------C TTTGATGTTG --CGTCGCAG TCACTCGTTG CTCGTCTGC- -TGCAAGGAC AGATCTATAT AACATATGCC CTGGATTCTT G-TACATT
TaMyc1.3 ---------G TGT----CCG --AATAGTAC TTTGCTGTTG C--GTCTGC- -TGCAAGGAC AGATCTATAG AACATATGCC CTGGATTCTT GGTACATT
TaMyc1.5 ---------C TTTGATGTTG --CGTCGCAC TCACTCGTTG CTCGTCTGC- -TGCAAGGAC AGATCTATAT AA----TGCC CTGGATTCTT GGTACATT
TaMyc1.2 CGAACAGTAC TTTGCTGTTG AACGTCGCAG TCACTCGTTC CTCGTCTGCG CTGCAAGGAC AGATCTATAT TA----TGCC CTGGATTCTT GGTGCATT
TaMyc1.4 CGAACAGTAC TTTGCTGTTG AACGTCGCAG TCACTCGTTC CACGTCTGCG CTGCAAGGAC AGATCTATAT TA----TGCC CTGGATTCTT GGTGCATT
