# Supplementary material for: Myc-like transcriptional factors in wheat: structural and functional organization of the subfamily I members
Source: BMC Plant Biol. 2019 Feb 15;19(Suppl 1):50. doi: 10.1186/s12870-019-1639-8 (PMC6393960; doi:10.1186/s12870-019-1639-8)
Supplement: Supplementary file 2 — Genetic similarity of promoter sequences of Myc-like genes subfamily I. Phylogenic tree was constructed in MEGA 7.0 with Neighbor-Joining method with 500 bootstrap replicates. Blue colour – the 1st group. Green colour – the 2nd group. (PPTX 37 kb) [file 12870_2019_1639_MOESM2_ESM.pptx]

## Slide 1
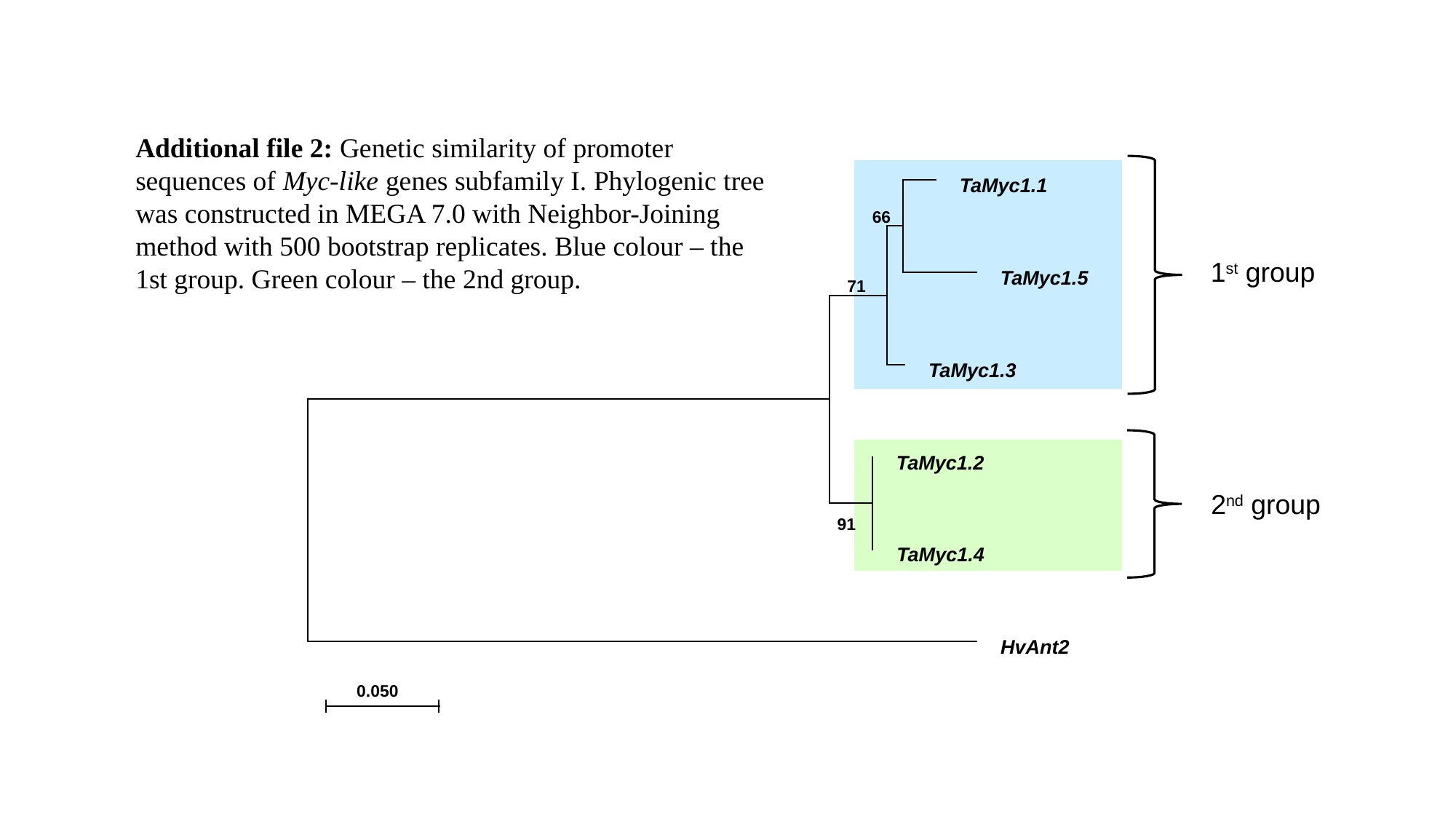

Additional file 2: Genetic similarity of promoter sequences of Myc-like genes subfamily I. Phylogenic tree was constructed in MEGA 7.0 with Neighbor-Joining method with 500 bootstrap replicates. Blue colour – the 1st group. Green colour – the 2nd group.
 TaMyc1.1
66
 TaMyc1.5
71
 TaMyc1.3
 TaMyc1.2
91
 TaMyc1.4
 HvAnt2
1st group
2nd group
0.050
