# Supplementary material for: Myc-like transcriptional factors in wheat: structural and functional organization of the subfamily I members
Source: BMC Plant Biol. 2019 Feb 15;19(Suppl 1):50. doi: 10.1186/s12870-019-1639-8 (PMC6393960; doi:10.1186/s12870-019-1639-8)
Supplement: Supplementary file 3 — Putative cis-acting regulatory elements identified in the Myc promoters. Promoter analysis was performed using New PLACE database. “+” – coding strand, “–” – template strand. (DOCX 27 kb) [file 12870_2019_1639_MOESM3_ESM.docx]

**Additional file 3:** Putative cis-acting regulatory elements identified in the *Myc* promoters. Promoter analysis was performed using New PLACE database. “+” – coding strand, “–” – template strand*.*

| **Motif** | **Sequence** | **Gene** | **Position (chain orientation)** | | **Description** | **References** |
| --- | --- | --- | --- | --- | --- | --- |
|  |  |  | “+” | “–” |  |  |
| TSS |  | *TaMyc-A1*  *TaMyc-A2*  *TaMyc-B1*  *TaMyc-D2*  *TaMyc-D1* | +1 (307)  +1 (350*)  +1 (314*)  +1 (354*)  +1 (304*) | | transcription start sites (TSS) were predicted by alignment with the wheat *TaMyc1* (*TaMyc-A1*) gene | 11 |
| RRE  E-box motif | CANNTG | *TaMyc-A1*  *TaMyc-A2*  *TaMyc-B1*  *TaMyc-D2*  *TaMyc-D1* | -248; -25  -269  -237; -32  -273  -233 | -248; -25  -269  -237; -32  -273  -233 | R response element (RRE); MYC recognition binding site | 7  8  9 |
| TATA-box | TATATAA  TATATtA  TATAgAA  TATATtA  TATATAA | *TaMyc-A1*  *TaMyc-A2*  *TaMyc-B1*  *TaMyc-D2*  *TaMyc-D1* | -32  -29  -39  -29  -29 |  | critical element for accurate transcription initiation around -30 of transcription start | 10 |
| G-box  ACGT motif | ACGT | *TaMyc-A1*  *TaMyc-A2*  *TaMyc-B1*  *TaMyc-D2*  *TaMyc-D1* | -266  -299; -73  -267; -240  -303; -73; -53  -263; -100 | -266  -299; -73  -267; -240  -303; -73; -53  -263; -100 | ACGT-containing elements required for etiolation-induced expression of the early responsive to dehydration gene | 15 |
| GTGA motif | GTGA | *TaMyc-A1*  *TaMyc-A2*  *TaMyc-B1*  *TaMyc-D2*  *TaMyc-D1* | -195; -120  -334; -221; -135  -300; -196; -121  -338; -221; -135  -296;-192; -117 | -220; -215; -65  -252; -247; -203; -64  -221; -216  -331; -252; -247; -203; -64  -289; -217; -212; -62 | motif found in the promoter late pollen gene | 13 |
| A core of TGAC-containing W-box | TGAC | *TaMyc-A1*  *TaMyc-A2*  *TaMyc-B1*  *TaMyc-D2*  *TaMyc-D1* | -232  -265  -233  -269  -229 | -221; -210; -66  -242; -183; -65  -222; -211  -242; -183; -65  -218; -207 | transcriptional repressor of the gibberellin signaling pathway in aleurone cells | 25 |
| DOF core | AAAG | *TaMyc-A1*  *TaMyc-A2*  *TaMyc-B1*  *TaMyc-D2*  *TaMyc-D1* | -123  -138  -124  -138  -120 | -84  -85  -71  -85  -81 | core site required for binding of Dof proteins | 14 |
| SORLIP | GGGCC | *TaMyc-A1*  *TaMyc-A2*  *TaMyc-B1*  *TaMyc-D2*  *TaMyc-D1* | -115  -130  -116  -130  -112 | -243  -276  -244  -280  -240 | sequences over-represented in light-induced promoters (SORLIP), *cis*-acting regulatory element involved in light responsiveness | 12 |
| GT1 consensus | GRWAAW  R=A/G  W=A/T | *-*  *-*  *TaMyc-B1*  *-*  *-* | -153 |  | *cis*-acting regulatory element involved in light responsiveness | 17 |
| MYB1 | WAACCA  W=A/T | *TaMyc-A1*  *TaMyc-A2*  *TaMyc-B1*  *TaMyc-D2*  *TaMyc-D1* | -150  -177  -163  -177  -147 | -150  -177  -177  -147 | MYB recognition site found in the promoters of dehydration-responsive genes and many other genes | 8 |
| ARE | TGGTTT | *TaMyc-A1*  *TaMyc-A2*  -  *TaMyc-D2*  *TaMyc-D1* |  | -150  -177  -177  -147 | *cis*-acting regulatory element essential for the anaerobic induction | 31 |
| MYCATRD22 | CTAACCA | *-*  -  *TaMyc-B1*  -  *-* | -164 |  | binding site for MYB in dehydration-responsive gene | 22  23 |
| ANAERO2 consensus | AGCAGC | *TaMyc-A1*  -  *-*  -  *TaMyc-D1* | -160  -157 |  | motif in promoters of anaerobic genes involved in the fermentative pathway (anaerobic set 1) | 24 |
| TC-rich repeats | GTTTTCTgAC | -  *TaMyc-A2*  -  *TaMyc-D2*  - |  | -183  -183 | *cis*-acting element involved in defense and stress responsiveness | 29  30 |
| Inr element | YTCANTYY  Y=C/T | *TaMyc-A1*  *TaMyc-A2*  *TaMyc-B1*  *TaMyc-D2*  *TaMyc-D1* | -178  -253; -204  -179  -253; -204  -174 |  | Initiator elements (Inr ) found in gene promoters without TATA-boxes; light-responsive transcription on Inr | 18 |
| CGTCA motif | CGTCA | *TaMyc-A1*  -  *TaMyc-B1*  -  *TaMyc-D1* | -222; -211  -223; -212  -219; 208 | -233 | *cis*-acting regulatory element associated with MeJA-responsiveness | 28 |
| GARE motif | AAACAGA | -  *TaMyc-A2*  *TaMyc-B1*  *TaMyc-D2*  *TaMyc-D1* | -239  -208  -233  -204 |  | gibberellin-responsive element (GARE) | 27 |
| W-box | TTGAC | *TaMyc-A1*  *TaMyc-A2*  *TaMyc-B1*  *TaMyc-D2*  *TaMyc-D1* |  | -210  -242  -211  -242  -207 | fungal elicitor responsive element, *cis*-regulatory elements recognized specifically by salicylic acid (SA)-induced WRKY DNA binding proteins | 32 |
| BIHD1 | TGTCA | -  *TaMyc-A2*  -  *TaMyc-D2*  - | -243  -243 |  | binding site of BELL homeodomain transcription factor | 19 |
| MBS | CAACTG | -  *TaMyc-A2*  -  *TaMyc-D2*  - | -269  -273 |  | MYB binding site (MBS) involved in drought-inducibility | 8 |
| E2F consensus sequence | WTTSSCSS  W=A/T  S=G/C | *TaMyc-A1*  *TaMyc-A2*  *TaMyc-B1*  *TaMyc-D2*  *TaMyc-D1* | -246  -279  -247  -283  -243 |  | E2F-DP-binding motif | 21 |
| CCAAT-box | CCAAT | *TaMyc-A1*  -  *TaMyc-B1*  -  *TaMyc-D1* | -249 |  | common *cis*-acting element in promoter and enhancer regions | 6 |
| CAAT-box | CAAAT | *-*  -  *TaMyc-B1*  -  *TaMyc-D1* | -237  -233 |  | common *cis*-acting element in promoter and enhancer regions | 6 |
| C-box | GACGTC | *TaMyc-A1*  *TaMyc-A2*  *TaMyc-B1*  *TaMyc-D2*  *TaMyc-D1* | -267  -300  -268  -304  -264 | -267  -300  -268  -304  -264 | one of ACGT elements; bZIP proteins binding site | 3  4  5 |
| DRE | RYCGAC  R=A/G  Y=C/T | *TaMyc-A1*  -  *TaMyc-B1*  -  *TaMyc-D1* | -270  -271  -267 | -270  -271  -267 | dehydration-responsive element (DRE) | 1  2 |
| Core CRT/DRE motif | GTCGAC | *TaMyc-A1*  -  *TaMyc-B1*  -  *TaMyc-D1* | -270  -271  -267 | -270  -271  -267 | C-repeat/ dehydration responsive element (CRT/DRE) motif; DNA binding is regulated by temperature | 26 |
| PHR1-binding sequence | GNATATNC | -  *TaMyc-A2*  -  *TaMyc-D2*  - | -294  -298 |  | motif in the upstream regions of phosphate starvation responsive genes | 20 |
| SURE core | GAGAC | -  *TaMyc-A2*  -  *TaMyc-D2*  - | -302  -306 |  | core of sulfur-responsive element (SURE) | 16 |

1. Xue G. P. Characterisation of the DNA‐binding profile of barley HvCBF1 using an enzymatic method for rapid, quantitative and high‐throughput analysis of the DNA‐binding activity //Nucleic Acids Research. – 2002. – Т. 30. – №. 15. – С. e77-e77.
2. Svensson J. T. et al. Transcriptome analysis of cold acclimation in barley Albina and Xantha mutants //Plant physiology. – 2006. – Т. 141. – №. 1. – С. 257-270.
3. Foster R., Izawa T., Chua N. H. Plant bZIP proteins gather at ACGT elements //The FASEB journal. – 1994. – Т. 8. – №. 2. – С. 192-200.
4. Izawa T. et al. The rice bZIP transcriptional activator RITA-1 is highly expressed during seed development //The Plant Cell. – 1994. – Т. 6. – №. 9. – С. 1277-1287.
5. Izawa T., Foster R., Chua N. H. Plant bZIP protein DNA binding specificity //Journal of molecular biology. – 1993. – Т. 230. – №. 4. – С. 1131-1144.
6. Shirsat A. et al. Sequences responsible for the tissue specific promoter activity of a pea legumin gene in tobacco //Molecular and General Genetics MGG. – 1989. – Т. 215. – №. 2. – С. 326-331.
7. Stålberg K. et al. Disruption of an overlapping E-box/ABRE motif abolished high transcription of the napA storage-protein promoter in transgenic Brassica napus seeds //Planta. – 1996. – Т. 199. – №. 4. – С. 515-519.
8. Abe H. et al. Arabidopsis AtMYC2 (bHLH) and AtMYB2 (MYB) function as transcriptional activators in abscisic acid signaling //The Plant Cell. – 2003. – Т. 15. – №. 1. – С. 63-78.
9. Hartmann U. et al. Differential combinatorial interactions of cis-acting elements recognized by R2R3-MYB, BZIP, and BHLH factors control light-responsive and tissue-specific activation of phenylpropanoid biosynthesis genes //Plant molecular biology. – 2005. – Т. 57. – №. 2. – С. 155-171.
10. Grace M. L. et al. Sequence and spacing of TATA box elements are critical for accurate initiation from the β-phaseolin promoter //Journal of Biological Chemistry. – 2004. – Т. 279. – №. 9. – С. 8102-8110.
11. Shoeva O. Y., Gordeeva E. I., Khlestkina E. K. The regulation of anthocyanin synthesis in the wheat pericarp //Molecules. – 2014. – Т. 19. – №. 12. – С. 20266-20279.
12. Hudson M. E., Quail P. H. Identification of promoter motifs involved in the network of phytochrome A-regulated gene expression by combined analysis of genomic sequence and microarray data //Plant Physiology. – 2003. – Т. 133. – №. 4. – С. 1605-1616.
13. Rogers H. J. et al. Functional analysis of cis-regulatory elements within the promoter of the tobacco late pollen gene g10 //Plant molecular biology. – 2001. – Т. 45. – №. 5. – С. 577-585.
14. Yanagisawa S., Schmidt R. J. Diversity and similarity among recognition sequences of Dof transcription factors //The Plant Journal. – 1999. – Т. 17. – №. 2. – С. 209-214.
15. Simpson S. D. et al. Two different novel cis‐acting elements of erd1, a clpA homologous Arabidopsis gene function in induction by dehydration stress and dark‐induced senescence //The Plant Journal. – 2003. – Т. 33. – №. 2. – С. 259-270.
16. Maruyama‐Nakashita A. et al. Identification of a novel cis‐acting element conferring sulfur deficiency response in Arabidopsis roots //The Plant Journal. – 2005. – Т. 42. – №. 3. – С. 305-314.
17. Terzaghi W. B., Cashmore A. R. Light-regulated transcription //Annual review of plant biology. – 1995. – Т. 46. – №. 1. – С. 445-474.
18. Nakamura M., Tsunoda T., Obokata J. Photosynthesis nuclear genes generally lack TATA‐boxes: a tobacco photosystem I gene responds to light through an initiator //The Plant Journal. – 2002. – Т. 29. – №. 1. – С. 1-10.
19. Luo H. et al. Up-regulation of OsBIHD1, a rice gene encoding BELL homeodomain transcriptional factor, in disease resistance responses //Plant Biology. – 2005. – Т. 7. – №. 5. – С. 459-468.
20. Rubio V. et al. A conserved MYB transcription factor involved in phosphate starvation signaling both in vascular plants and in unicellular algae //Genes & development. – 2001. – Т. 15. – №. 16. – С. 2122-2133.
21. Vandepoele K. et al. Genome-wide identification of potential plant E2F target genes //Plant Physiology. – 2005. – Т. 139. – №. 1. – С. 316-328.
22. Abe H. et al. Role of Arabidopsis MYC and MYB homologs in drought-and abscisic acid-regulated gene expression //The Plant Cell. – 1997. – Т. 9. – №. 10. – С. 1859-1868.
23. Busk P. K., Pages M. Regulation of abscisic acid-induced transcription //Plant molecular biology. – 1998. – Т. 37. – №. 3. – С. 425-435.
24. Mohanty B. et al. Detection and preliminary analysis of motifs in promoters of anaerobically induced genes of different plant species //Annals of Botany. – 2005. – Т. 96. – №. 4. – С. 669-681.
25. Zhang Z. L. et al. A rice WRKY gene encodes a transcriptional repressor of the gibberellin signaling pathway in aleurone cells //Plant Physiology. – 2004. – Т. 134. – №. 4. – С. 1500-1513.
26. Xue G. P. The DNA‐binding activity of an AP2 transcriptional activator HvCBF2 involved in regulation of low‐temperature responsive genes in barley is modulated by temperature //The Plant Journal. – 2003. – Т. 33. – №. 2. – С. 373-383.
27. Skriver K. et al. cis-acting DNA elements responsive to gibberellin and its antagonist abscisic acid //Proceedings of the National Academy of Sciences. – 1991. – Т. 88. – №. 16. – С. 7266-7270.
28. Thijs G. et al. A Gibbs sampling method to detect overrepresented motifs in the upstream regions of coexpressed genes //Journal of Computational Biology. – 2002. – Т. 9. – №. 2. – С. 447-464.
29. Ozkaynak E. et al. The yeast ubiquitin genes: a family of natural gene fusions //The EMBO journal. – 1987. – Т. 6. – №. 5. – С. 1429.
30. Gausing K., Jensen C. B. Two ubiquitin-long-tail fusion genes arranged as closely spaced direct repeats in barley //Gene. – 1990. – Т. 94. – №. 2. – С. 165-171.
31. Walker J. C. et al. DNA sequences required for anaerobic expression of the maize alcohol dehydrogenase 1 gene //Proceedings of the National Academy of Sciences. – 1987. – Т. 84. – №. 19. – С. 6624-6628.
32. Yu D., Chen C., Chen Z. Evidence for an important role of WRKY DNA binding proteins in the regulation of NPR1 gene expression //The Plant Cell. – 2001. – Т. 13. – №. 7. – С. 1527-1540.
