# Supplementary material for: Myc-like transcriptional factors in wheat: structural and functional organization of the subfamily I members
Source: BMC Plant Biol. 2019 Feb 15;19(Suppl 1):50. doi: 10.1186/s12870-019-1639-8 (PMC6393960; doi:10.1186/s12870-019-1639-8)
Supplement: Supplementary file 4 — Coleoptile colour of selected wheat samples at the fifth day after germination. (PPTX 459 kb) [file 12870_2019_1639_MOESM4_ESM.pptx]

## Slide 1
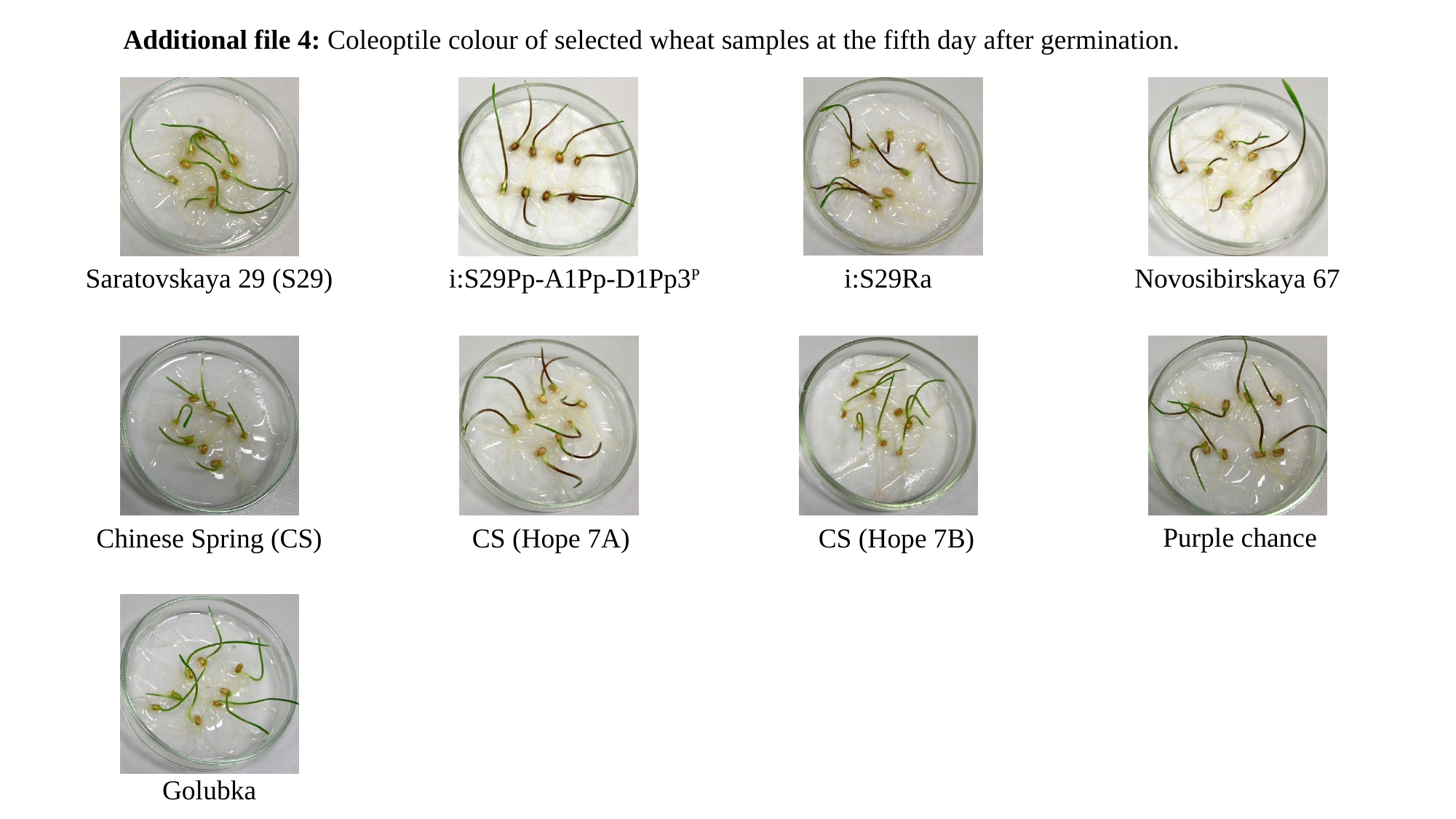

Additional file 4: Coleoptile colour of selected wheat samples at the fifth day after germination.
Saratovskaya 29 (S29)
i:S29Pp-A1Pp-D1Pp3P
i:S29Ra
Novosibirskaya 67
Purple chance
Chinese Spring (CS)
CS (Hope 7A)
CS (Hope 7B)
Golubka
