# Supplementary material for: Myc-like transcriptional factors in wheat: structural and functional organization of the subfamily I members
Source: BMC Plant Biol. 2019 Feb 15;19(Suppl 1):50. doi: 10.1186/s12870-019-1639-8 (PMC6393960; doi:10.1186/s12870-019-1639-8)
Supplement: Supplementary file 5 — The results of bisulfite sequencing in genotypes of near isogenic lines, differ in allelic state of TaMyc-A1: lines S29 and i:S29Pp-A1Pp-D1Pp3P. First and second exons are marketed with yellow and green colour, respectively. Blue – unmethylated CpG sites, red – start-codon (ATG site). (DOCX 13 kb) [file 12870_2019_1639_MOESM5_ESM.docx]

**Additional file 5:** The results of bisulfite sequencing in genotypes of near isogenic lines, differ in allelic state of *TaMyc-A1*: lines S29 and i:S29Pp-A1Pp-D1Pp3^P^. First and second exons are marketed with yellow and green colour, respectively. Blue – unmethylated CpG sites, red – start-codon (ATG site).

**>S29_TaMyc-A1**

TGTTGGAATATACGTATAAGATTTTCGTAGTTATATTTTAATTATTAGATTAAGTATAGTTTTTTTGGTAGAACGTTTTTGTATTATTATTGTTGATATTATAATTGTATCGGTAGGTCGTGTTTTTTTAGGCGTCGTCGACGTCGATTATTTTGCGTTAATTGGTTTTACGAATGATTTATTTCGTTATTTTATCGTTAAGTAGAAATCGGTGAGGATTTCGTGTATTTTAGTTTAGTTTTATTAAGTAGCGAGAAAATTAAGTAGGTATTATATTAGTTAGAAAGTGATGGGTTTAATTGTATGTTAGTGTAGTATAGTATTTTGATGTTGCGTCGTAGTTATTCGTTGTTCGTTTGTTGTAAGGATAGATTTATATAATATATGTTTTGGATTTTTGTATATTGTATATATAGCGGTTTTATTTTTTTTTTCGGACGATAGGTTGGTTTTTGAGTTTTTTGGTTTTTATAGTTTAAGTAAATTTTTAATAGTTTTTTAGTTGGAAGTTTTTTTTTTATTTATGGGTTATGGTTAGTTTATTTTTTAGTTTTTTAGTTAGCGTTAGTGTAATAGAGTAAAGAGATTTGATTAAATTAGTTAAGAAGATGTATTATGTTTATTATGAATAGTAGAAATGAGGTTATAGTTTTATTGATTTGTTTATTTTTTGTTTTTTGGTTTGTTTTAATTTTATTTTGTTTGTATGTATGTATATGAAGGAAGGAAATAGTGTGGTAATGGCGTT

**>i:S29Pp-A1Pp-D1Pp3^P^_TaMyc-A1**

TGTTGGAATATACGTATAAGATTTTCGTAGTTATATTTTAATTATTAGATTAAGTATAGTTTTTTTGGTAGAACGTTTTTGTATTATTATTGTTGATATTATAATTGTATCGGTAGGTCGTGTTTTTTTAGGCGTCGTCGACGTCGATTATTTTGCGTTAATTGGTTTTACGAATGATTTATTTCGTTATTTTATCGTTAAGTAGAAATCGGTGAGGATTTCGTGTATTTTAGTTTAGTTTTATTAAGTAGCGAGAAAATTAAGTAGGTATTATATTAGTTAGAAAGTGATGGGTTTAATTGTATGTTAGTGTAGTATAGTATTTTGATGTTGCGTCGTAGTTATTCGTTGTTCGTTTGTTGTAAGGATAGATTTATATAATATATGTTTTGGATTTTTGTATATTGTATATATAGCGGTTTTATTTTTTTTTTCGGACGATAGGTTGGTTTTTGAGTTTTTTGGTTTTTATAGTTTAAGTAAATTTTTAATAGTTTTTTAGTTGGAAGTTTTTTTTTTATTTATGGGTTATGGTTAGTTTATTTTTTAGTTTTTTAGTTAGCGTTAGTGTAATAGAGTAAAGAGATTTGATTAAATTAGTTAAGAAGATGTATTATGTTTATTATGAATAGTAGAAATGAGGTTATAGTTTTATTGATTTGTTTATTTTTTGTTTTTTGGTTTGTTTTAATTTTATTTTGTTTGTATGTATGTATATGAAGGAAGGAAATAGTGTGGTAATGGCGTT
